# Bethlem Hospital
*Physicians: Dr. Monro; Sir A. Morison. Surgeon: W. Lawrence, Esq. Resident Medical Officer: Dr. W. Wood.


**Published:** 1851-04-01

**Authors:** 


### BETHLEM HOSPITAL.*

(WITH AN ILLUSTRATION.)

This ancient institution (one of the first, if not the oldest, asylums for lunatics in Europe) was founded in 1247, during the reign of Henry III., by Simon Fitzmary, a sheriff of London, at Bishopsgate, when it was designated the Priory of Bethlehem. At the dissolution of the religious houses, the property was seized by Henry VIII., who in 1547 granted the Priory, with all its revenues, to the Corporation of London; from which time, it was appropriated as a hospital for the cure of lunatics. Owing to the limited income the institution possessed, the usefulness of this charity was necessarily circumscribed, until the year 1675; when, by the liberality of benevolent individuals, a splendid building was erected in Moorfields for the reception of 152 insane patients. Tradition reports that the design was taken from the Tuileries; which so incensed the "grande monarque," Louis XIV., when he heard his regal château had been made the model of a madhouse in London, that he ordered a plan of St. James's palace to be taken for offices of the meanest nature. Over the gates of the old hospital, constructed in Moorfields, the two celebrated statues were placed of

---

* Physicians: Dr. Monro; Sir A. Morison. Surgeon: W. Lawrence, Esq. Resident Medical Officer: Dr. W. Wood.

A committee of governors, along with the physicians, meet at the hospital every Friday forenoon, for the reception of new patients, who are generally admitted, if proper objects of charity, excepting the following cases:—

1. Those lunatics who are possessed of property sufficient for their decent support in a private asylum.
2. *Those who have been insane for more than twelve months.*
3. Those who have been discharged *uncured* from any other hospital for the reception of lunatics.
4. Female lunatics who are with child.
5. Lunatics in a state of idiocy, *afflicted with paralysis*, or with epileptic or convulsive fits.
6. Lunatics having the venereal disease or the itch.
7. Lunatics who are blind, or so weakened by age, or by disease, as to require the attendance of a nurse, or to threaten the speedy dissolution of life, or who are so lame as to require the assistance of a crutch, or a wooden leg.

Previous to the admission of any individual into Bethlem Hospital, a certificate of insanity, signed by a legally qualified medical practitioner stating that the person, for whom application is made, is a lunatic, must be presented to the committee; and as only one medical gentleman is required to sign that important document, he must mention the nature of his qualification, and the college from whence he obtained a diploma to practise, according to the subjoined form:—

| | | |
|---|---|---|
| ____________________ | Fellow | Of the Royal College of Physicians, London. |
| ____________________ | Licentiate | |
| ____________________ | | Graduate in Medicine of the British University of ____________ |
| ____________________ | Fellow | Of the Royal College of Surgeons of England. |
| ____________________ | Member | |
| ____________________ | | Apothecary duly authorized to practise by the Apothecaries' Company, London. |

The medical practitioner who signs this certificate, is requested to write his name against the proper denomination.

The commissioners in lunacy might adopt a similar proceeding with advantage, and require all parties signing the medical certificate of insanity, to give in full the nature of their qualification, as now followed at Bethlem Hospital, which institution is not within the commissioners' jurisdiction.

Melancholy and Raving Madness, sculptured by Caius Gabriel Cibber, the father of Colley Cibber the comedian; these are still seen in the vestibule of the modern structure in St. George's Fields, Lambeth.

Notwithstanding the addition of wings to the original building in Moorfields, it was found inadequate to the increasing claims made for admission within its walls; hence, in 1812, the first stone of the present edifice was laid, to contain 198 lunatics. In August, 1815, the new hospital, being completed, was opened for the reception of patients; nevertheless, like the old institution in the city of London, it was soon found inadequate for the wants of an increasing population. The charity having now, however, ample funds at their disposal, the governors resolved to build additional dormitories for 166 inmates; these were accordingly commenced in July, 1838, and finished about two years afterwards. Although the accommodation afforded in the new hospital was thus made considerably greater than previously, two other wings, chiefly for convalescent patients, were added in 1845; whereby, also, a better classification of the lunatics could be accomplished. Along with these additions, the central dome which now towers above all the neighbouring buildings, was erected, in 1848, to serve as a chapel for patients and residents.

In consequence of the various additions thus made to Bethlem Hospital, the institution is capable of accommodating 450 inmates; although the average number of patients, usually under treatment, seldom exceeds 400, including the criminal and the incurable lunatics.

The medical staff of the hospital consists of two physicians, viz., Dr. Monro and Sir Alexander Morison; one surgeon, Mr. Lawrence; and a resident medical officer, Dr. Wood, who alone resides on the premises. Consequently, during twenty-two out of the twenty-four hours of every day, on that gentleman rests the responsibility of superintending the medical treatment ordered by his superior officers, for so large a population; as there are neither house-surgeons nor resident pupils, as in many foreign lunatic asylums, or even some English establishments.

Bethlem is a hospital for the cure of mental diseases, not an asylum for lunatics. Although a limited number of incurable insane patients now reside within its walls, in consequence of a valuable estate in Lincolnshire having been left for the express purpose of maintaining incurable and dangerous lunatics, a large proportion of the patients under treatment are recent cases, whose mental malady has not been of long continuance; and no lunatic is admitted who has continued more than one year insane. In consequence of this regulation, the annual number of admissions is generally considerable: and the character of the maladies, affecting the patients under treatment, is more acute, and often exhibits very different types of disease from those usually manifested by the inmates of county lunatic asylums.

During the year 1850, the number of curable lunatic patients admitted into Bethlem Hospital, was 344, of whom 135 were males, and 209 females; thus giving a preponderance of 74 in the latter sex, or 54 per hundred. The number of patients discharged cured during the same period, amounted to 197, consisting of 74 men and 123 women; being at the rate of 57 per cent. of cures, which forms a high ratio, and is considerably beyond the amount discharged convalescent in previous years. Amongst the curable patients, the number of deaths was 31, which exceeded the usual average. This arose chiefly from the circumstance, that last year many individuals were received into the house almost in a dying state, through motives of charity. Much relief was thus given to the relatives and families of the afflicted sufferers, as well as comfort to the patients themselves. Besides the 31 deaths now enumerated, 4 incurable and 6 criminal lunatics died in 1850; so that 41 is the total number of fatal cases during the past year.

Speaking generally, 13 of the total deaths were classified as the immediate consequence of disease of the head and nervous system, 3 of which arose from general paralysis. This fact deserves notice, seeing that the malady is considered of less frequent occurrence in England than on the continent; especially if London be compared with Paris. Sixteen patients died from affections of the thoracic organs; of whom 6 were carried off by phthisis, and 3 by that rare form of disease,—except amongst the insane,—gangrene of the lungs. Respecting this unusual morbid change of structure in the respiratory organs, it may be interesting to mention, that the three instances all occurred in male patients; and as two had been only four weeks in the hospital, the other about eight months, the mental disease, consequently, had not likely been of long standing; whilst neither of the patients were old men, one being 45, another 41, and the third in his thirtieth year. *Exhaustion* is reported as the cause of death in nine cases. This expression is too indefinite; and might, we think, be generally superseded by more precise phraseology in a hospital report regarding disease amongst lunatics.

"Another interesting feature connected with the insane patients admitted during the last year into Bethlem Hospital, is *the apparent cause which produced mental disease*. This constitutes an important subject of investigation in all complaints, but especially in those of the mind; and as minute inquiries are always made respecting the above point, when patients enter this institution, much curious and instructive information is often obtained.

"As it would be incompatible with the present brief sketch of Bethlem Hospital, to enumerate every particular cause producing insanity in the 344 patients admitted during the year 1850, a short summary, with a few illustrations, will suffice to show the chief influences which appeared to produce the attacks of mental alienation. Speaking generally, one half of the cases, in both sexes, were ascertained to have arisen from moral causes. Anxiety appearing the most frequent influence; 26 women and 22 men being so classed. Grief at the death of friends was reported to have produced insanity in 18 females, but in only 2 men. Love caused madness in 12 females; but not a single instance occurred amongst the male patients; whilst religion was assigned as the apparent cause of mental disease in 8 women and in 6 men. Various other moral agents might be also enumerated, which, however instructive, would be tedious to particularize; nevertheless, one or two curious examples of the powerful effect often produced upon weak human minds by transitory influences may be mentioned, as they show how easily the mental faculties are disordered, and sometimes even completely upset, by temporary impressions, doubtless strongly acting at the time upon the nervous system, and a susceptible constitution. Thus, two men became mad from the fear of being attacked by cholera, and one from political excitement. Two women, on the other hand, were deprived of reason from living with insane persons; one from attending a singing-class; another female lost her senses from terror at the revolutionary disturbances in Paris; whilst a fifth became insane from the excitement of travelling, for the first time, by railway.

"Amongst the physical causes producing insanity, intemperance was reported as the most frequent, especially in men; nine cases having been met with in that sex, while eight occurred amongst the female patients, although the latter were more numerous. Bodily illness produced madness in 13 women, but only five instances occurred amongst the male inmates. Eight cases of puerperal insanity were admitted, and three arose from lactation; four from change of life; two from uterine disturbance; and one female was reported to have gone mad from her recent marriage. Again, amongst the male patients, four were stated to have become lunatics from solitary sexual excess; three from exposure to a hot sun; one from the disappearance of an accustomed eruption; another in consequence of an attack of cholera; whilst one poor fellow became insane from severe sea-sickness. Lastly, amongst the whole 135 male lunatics admitted, 39 cases were ascertained to have hereditary tendency to insanity, which makes about 29 per cent.; whereas, amongst the 209 female patients, 67 exhibited hereditary tendency to mania, being at the rate of 32 per cent. on the admissions; thus proving that mental diseases, besides being more frequent in women, are likewise transmitted to the offspring in a higher proportion amongst them than in the opposite sex.

"In addition to the above summary of the chief causes which apparently produced mental alienation in the various individuals admitted into Bethlem Hospital, it may be interesting to mention, especially as the frequently severe character of the cases is thereby manifested, that more than half the male patients, or 76 of the 135 admitted, were classed as violent cases, most being likewise dangerous either to others or themselves. Farther, amongst the 209 female lunatics also admitted during 1850, nearly one-half, or 100, were enumerated in the same category, of whom 90 were decidedly dangerous lunatics. It is also worthy of special notice, that 58, or nearly one half of the male patients received, were classed as suicidal; of whom 14 had actually endeavoured to destroy themselves previous to their admission. Again, amongst the 209 female lunatics, almost one-half, or 103, were ascertained to be suicidal patients, of whom 28 had attempted suicide prior to becoming inmates of this establishment.

The above interesting facts are now briefly mentioned, not only to show the forms of mental disease generally met with in the curable wards of Bethlem Hospital, but also to prove, that unlike most public receptacles for lunatics, particularly county asylums, this institution is, in great part, appropriated for the reception of recent cases of insanity, and therefore constitutes a hospital for the cure of insane patients, rather than a refuge for the hopeless victims of that terrible calamity. Hence, those who have remained more than one year within its walls, without receiving benefit, are then discharged as uncured, in order to be replaced by others more recently attacked with insanity. Although relatives may be sometimes inconvenienced by having their afflicted friends thus sent home before they are convalescent, and with perhaps slighter hopes of improvement than existed previous to admission, it ought to be always remembered that, during one year at least, and occasionally even for fifteen or eighteen months, such patients have been maintained and treated gratuitously; and although they no longer receive the benefits of this charity, other fellow-creatures equally, or even more severely afflicted, then occupy the recently evacuated dormitories. By this arrangement, a larger number of lunatics are annually received, than could be otherwise admitted; and in this way the utility of the institution becomes more extensively diffused. As every English county now has, or ought to possess, an asylum for the reception of its lunatic poor, the regulations in force at Bethlem Hospital render its benefits more generally useful, notwithstanding instances may occasionally occur where a rigid adherence to the spirit of the rules may prove inconvenient to individuals.

Bethlem, being an hospital appropriated chiefly for recent cases of insanity, whilst the criminal and incurable lunatic patients also furnish numerous illustrations of chronic and protracted instances of mental disease, supplies an excellent field for the study and investigation of mania in every variety. Until recently, medical students could nowhere else in the metropolis obtain clinical instruction and practical knowledge respecting the treatment of insanity, except at this hospital. But even here, owing to the very high fee charged for permission to attend the physicians, and as only three pupils could be entered at the same period, the doors of the institution became almost wholly shut to the general body of the profession; so that few students, excepting those specially intending to treat mental diseases, were induced to avail themselves of the means of instruction here afforded to them.

About nine years ago, Dr. Webster, then a governor of the hospital, brought the subject of allowing medical pupils greater facilities, when attending in the wards of the institution, before the managing authorities. Considerable discussion ensued upon his proposition, which it now seems wholly unnecessary to detail. Nevertheless, the agitation, thus commenced, ended ultimately in throwing open the portals of this large charity to all medical students who might feel anxious to obtain knowledge respecting the treatment of insanity, and to witness the practice of the physicians; the admission fee for each term—extending to about four months—being reduced to three guineas; whilst, in the summer season, clinical lectures are given by one of the medical officers. During last year, Dr. Monro lectured to the pupils, of whom generally about ten attended very regularly. In the ensuing summer term, Sir A. Morison will probably undertake that duty, as he did during 1849. Hence, no professional person can complain that he is unable to learn the nature, symptoms, treatment, and pathology of mania; for not only Bethlem Hospital, but St. Luke's, and the Hanwell Asylum, are now accessible to properly qualified applicants.

Notwithstanding these facilities for studying insanity, the numbers likely to take advantage of the ample opportunities at present available, will not, probably, be numerous, until the licensing medical corporations require from all aspirants for their honours, practical proofs that they are conversant with diseases of the mind as well as of the body. Formerly, it was sometimes said to be useless to insist on candidates for diplomas from the Colleges possessing any knowledge of mental diseases, as no place was open for obtaining such knowledge. Now, others even assert it to be unnecessary to enter hospitals for the insane, as the Colleges do not examine on insanity, and there is plenty to learn besides. Such reasoning may be agreeable to the inactive student; but it only shows the necessity which exists of medical colleges, and public bodies, altering their system, especially in reference to medical officers entering the army and the East India service. Indeed, a rumour prevails, that an intention exists of requiring from the future surgeons appointed to the Honourable Company, a certificate of having attended at a public lunatic institution, where they have had proper opportunities of acquiring an adequate knowledge of the management of mental diseases. This would be a very judicious proceeding; and however necessary for those filling military medical appointments, it is still more essential for civil practitioners; especially as they are liable, at any time, to be called upon to attend cases of madness, and sometimes on an emergency; which duty they should be as much qualified to undertake, as to treat any severe attack of fever, an accident, or accouchement. Besides this, if both legally and medically conversant with the subject of insanity, the physician or surgeon would be then fully prepared to enter courts of justice, when called as a witness in cases *de lunatico inquirendo.* He would then be able both to speak confidently, as well as scientifically, respecting the symptoms and condition of the individual under investigation, instead of being sometimes brow-beaten by astute barristers, who even consider it a great triumph if they can invalidate the evidence of "doctors," by making them give obscure answers to difficult questions, and so puzzle the court and jury through mystifications. This an experienced practitioner ought to be fully qualified to clear up or explain, with credit to himself, and with advantage to the afflicted lunatic.

Elaborate and varied statistical data are annually prepared, containing many instructive particulars respecting the curable patients under treatment, during the year. These tables were commenced, in their present form, in 1843, by Dr. Webster, who arranged the plan adopted, and compiled the different returns for that and the succeeding year, when they were printed for the use of the governors, along with the Report of the General Committee of Management. Exactly parallel statements have been drawn up regularly ever since they were commenced, more recently by the resident medical officer, Dr. Wood, and these are now distributed to the governors along with the Physicians' Annual Report. As it would be tedious to enumerate the different subjects embraced in these official documents—amounting to thirty-five in number,—the reader need be only now informed, that they contain, amongst other particulars, the condition of the inmates admitted, their former occupations, the assigned causes of their disease, and whether dangerous, violent, or suicidal patients. The symptoms and treatment, also the apparent cause of death, the numbers discharged uncured, or sent home convalescent, are carefully recorded. The influence which age, season of the year, duration of the attack previous to admission, the patients' domestic condition, degree of education, nativity, and religious persuasion, have exercised upon the disease, are likewise registered; besides other data of much value to the student and medical practitioner. As these tables have now been continued, on the same model, for eight consecutive years, and already form a large collection of important information, they will every day become of greater value to psychologists.—*Lon. Jour. of Med.*

